# Buffalo Academy of Medicine

**Published:** 1900-12

**Authors:** F. W. McGuire


### BUFFALO ACADEMY OF MEDICINE.

*Section on Pathology, September 18, 1900.*

REPORTED BY F. W. McGUIRE, M. D., Secretary.

THE first meeting of the year of the section was held at the Academy rooms, Public Library Building, Tuesday, September 18, 1900, twenty-seven Fellows being present.

Dr. EUGENE A. SMITH in the chair, called the meeting to order at 9 o'clock p.m.

The minutes of the previous meeting weie read and approved.

The essayist of the evening was Dr. H. G. MATZINGER, who read a paper entitled, Blood Examinations. (See page 319.)

Dr. ALBERT E. WOEHNERT said he agreed that great care should be used in technique, as errors were usually attributed to carelessness in this direction. He also thought that blood examinations had proved to be of more value from a diagnostic rather than a therapeutic standpoint. He considered the specific gravity method the most accurate in estimating hemaglobin.

Dr. JULIUS ULLMAN said he ordinarily made a rough examination, first, by placing some fresh blood on a slide, and if he found over six or seven polymorphonuclear white cells in a fluid he then made a more accurate examination as to leucocytosis.

Dr. CHAS. S. JEWETT thought we ought to be conservative and not to consider the examination of the blood alone, but to consider it in connection with the symptoms of the case.

Dr. A. L. Benedict uses the centrifuge in estimating hemaglobin. He uses both tubes, and if there is a difference of more than 1 per cent, he discards the test and makes another attempt. He does not like the specific gravity method. To be thoroughly accurate it is necessary to estimate the amount of iron in the blood.

Dr. De Lancey Rochester cited an obscure case in which the diagnosis was proved to be malaria by the blood examination. He said, in cases of diminished hemoglobin, iron should be used, but where there is a diminution in the number of red cells, arsenic should be used. He further cited a case in which a diagnosis of pus in the abdomen was made through the examination of the blood.

Dr. J. W. Grosvenor wished to know if, in Dr. Rochester's case, there was any other condition present which could account for leucocytes.

Dr. M. Hartwig cited a case and spoke of the advantages of blood examination.

Dr. Frost, Buffalo State Hospital, exhibited a specimen of rupture of the heart.

Meeting adjourned at 10.30 p.m.

---

*Section on Medicine, October 9, 1900.*

Reported by JULIUS ULLMAN, M. D., Secretary.

The first regular meeting of the section on medicine for the year was held in the Buffalo Public Library Building, Tuesday evening, October 9, 1900, the chairman, Dr. Eli Long presiding.

Dr. William G. Spiller, professor of nervous diseases, Philadelphia Polyclinic, read a paper on The involvement of the nervous system in malaria. He reported a case of malaria presenting the symptoms of disseminated sclerosis, and showed microscopical slides of the tissue.

He referred to a number of clinical papers in which monoplegia, paraplegia, hemaplegia, aphasia, convulsions and tremor were reported as occurring in persons with malaria. The diagnosis in these cases was frequently made only from the results obtained by the administration of quinine, and syphilis was not always excluded. It is probable, however, that these disturbances may result from malaria. The malarial parasite has been found within the central nervous system by some observers, but never previously in a case with the symptoms of disseminated sclerosis, and the real nature of this so-called malarial disseminated sclerosis has hitherto been unknown. Dr. Spiller referred to the various pathological conditions found in the nervous system in cases of malaria, and reported a case in which the symptoms were: a marked intention, tremors of the left upper limb, transitory hemiparesia, diplopia, marked ataxia of the left lower limb, marked vertical nystagmus, distinctly scanning speech and exaggerated tendon reflexes on the right side. The man, a sailor, died after a severe attack of diarrhea, probably of malarial origin.

In the microscopical examination every capillary of the central nervous system was found plugged with pigmented malarial parasites of the estivo-autumnal form. An area of slight sclerosis was found in the outer part of the middle third of the left crusta and the right crossed pyramidal tract was slightly but distinctly sclerotic. The most probable cause for this slight sclerosis of only one motor tract was probably small hemorrhage of early formation. This view was confirmed by the presence of numerous small recent hemorrhages and altered blood pigment with the central nervous system. The only apparent cause for the hemorrhages was found in the presence of the malarial parasites. No area of disseminated sclerosis was seen. The case shows that the symptoms of disseminated sclerosis occurring from malaria probably are the result of vascular lesions. It is not improbable that similar symptoms occurring rapidly after infectious diseases, such as typhoid, may sometimes be the result of poisoning of the nerve centers and not of true disseminated foci sclerosis.

### DISCUSSION.

Dr. Charles Cary: The study of malaria is becoming very important to us, because many cases are bound to come to us now because of our new possessions in the tropics. Malaria in Buffalo is imported. I have seen few, if any, cases of malaria that are not imported. There are no anapholes in Buffalo.

Dr. Cary urged the theory that the transitory symptoms (nervous) in malaria are not due so much to a degeneration as to a fulness of the vessels with the blood corpuscles and the parasites. This favors an arrest of circulation and a temporary thrombosis. The healthy corpuscles clear out this temporary thrombosis and this may account for symptoms varying from a few hours to days. The cause of graver symptoms is to be looked for in the location of the blocking of the circulation.

Dr. James W. Putnam maintained that malaria is the cause of many nervous diseases, such as disseminated sclerosis, hemiplegia, multiple neuritis and the like. He stated that lately it has been suggested that infectious diseases may be contracted for the cure of nervous diseases and because some few epileptics who contracted malaria are apparently benefited, some are sending epileptics into malarial districts.

Dr. H. U. Williams questioned the author as to the toxic action of the malarial poison producing degenerative changes in the ganglion cells.

Dr. Marcel Hartwig asked concerning the pathological anatomy of supra-orbital neuralgia following malaria. He seems certain that such exists and has seen it in patients in whom, however, no parasites were sought.

Dr. Arthur Bennett stated that he observed hyperplasia in malarial supra-orbital neuralgia and observed also that one eye lies higher than the other.

Dr. Gaylord asked concerning the fibrin formation in the thrombi.

Dr. E. E. Blaauw asked why the sclerosis in Dr. Spiller's case could not just as well be attributed to the early history of syphilis in the case.

Dr. Marshall Clinton reported the case of an officer in Cuba who while riding his horse was seized with a chill and afterward became delerious. The first attack lasted eighteen days. On the twenty-first day after the first attack the patient had another severe chill and died in six hours. Necropsy showed a serous apoplexy. The lateral ventricle was distended, there were petechial hemorrhage of brain. No malarial organisms were demonstrated.

Dr. W. G. Spiller in conclusion, stated that all vessels thrombosed in this case must have been in this condition but a short time before death, for if this persisted it would show a degeneration of nerve cell. This case was not diagnosticated or treated as malaria during life. Dr. Spiller could not state that toxemia was a factor in this case. He found no change in the nerve cells.

Cases are reported of supraorbital neuralgia, due to malarial infection which improve on quinine, but no parasite is found in the blood. He quoted Thayer, who does not believe this neuralgia to be due to malaria. He did not examine the sections for fibrin, so that cannot answer as to fibrin formation. As to syphilis in this case, he could not detect any of the changes of syphilis in the vessel walls of the central nervous system. In this case there was only a very slight sclerosis, due to a former hemorrhage.

On motion of Dr. Wm. C. Krauss, seconded by Dr. Hartwig, a vote of thanks was tendered to Dr. Spiller.

Number in attendance, 50. Adjourned 10.30 p.m.

---

*Section on Ophthalmology, Otology, Rhinology and Laryngology, October 22, 1900.*

Reported by GEORGE F. COTT, M. D., Secretary.

Meeting was called to order at 9 p.m. Dr. F. W. Hinkel in the chair. Minutes of previous meeting adopted.

Dr. E. E. Blaauw presented a case of a girl aged 20, who had total absence of abduction. The affection is of long standing, although the patient had never noticed any trouble. She consulted Dr. Blaauw on account of headache. The condition may be congenital, or acquired in early youth. The trouble may be muscular or nervous; if the latter, it is neither the central or peripheral.

Dr. Snow reported a case of retropharyngeal adenitis, and two cases of retropharyngeal abscess occurring in young children.

DISCUSSION.

Dr. E. A. Smith.—The essayist has covered the subject so carefully and thoroughly, that it is hardly necessary to say any more. My impression in that case was somewhat different. I would follow a different procedure and give chloroform next time. Of course, that is theoretically easier to carry out than it seems. I could not detect fluctuation, when I examined the patient. I hesitated somewhat and thought it best to wait till morning. I did not feel anxious to operate that night. No doubt the mouth-gag had something to do with the death. If I felt certain of abscess, I would have plunged a knife in without hesitation. This case was either one of retropharngeal or postmediastinal abscess. I have only met one case of retropharyngeal abscess before, and that was post mortem, in which no diagnosis was made. It was found to have been due to caries. If I should meet another similar case, I should anesthetise, do a tracheotomy, and then open the abscess. I want to see where aspirator or bistoury is going. By feeling, the bulging seemed to be more like sarcoma, considering also other symptoms. Death was probably due to septicemia.

Dr. CHAUNCEY P. SMITH.—I think Dr. Snow covered the ground thoroughly. We must consider the acuteness and chronicity of these abscesses. Acute abscesses get well while the chronic or tubercular take many months to improve. Postmediastinal abscess may be extremely hard to palpate, being so low, and then there is the danger of mediastinal infection, owing to the fact that it is better to open from the outside when there is no danger to it; a grooved director is to be used, which makes the operation quite safe, or all the vessels and nerves may be pulled forward and then the abscess opened that way.

Dr. A. A, JONES.—I would like to ask what percentage of cases of retropharyngeal abscess are connected with caries of the vertebra.

Dr. F. W. HINKEL. —The report of these cases calls to my mind the case of a child ten months of age, in which there was distinct pointing of the abscess externally after two weeks sickness. Dr. Park opened externally. Dr. Hopkins also saw the case. There was slight hemorrhage in the throat, back of the tonsils blood was trickling down the pharynx. The wall was so thin that one could distinctly feel the touch of the thumb and finger. I recommended the replacing of the dressings. The child was very feeble and poorly nourished and finally died. The abscess pointed just below the ear. Chloroform is recommended by some authorities. There is certainly no theoretical objection. It is interesting to note the association of adenitis with nasal inflammation.

Dr. SNOW.—Regarding the percentage of cases, Bokai saw 600 cases, of which 120 were due to caries; the remainder were idiopathic, due to infection from the throat. My paper was on idiopathic abscesses.

The mortality of idiopathic cases is only 4½ per cent. by Bokai. The operation of opening is very quickly done, he being very expert, having had more experience than any living man, and had no accident reported; in abscess low down, however, I can readily see the danger.

Then followed the paper by Dr. Ullman on

THE TONSIL AS A PORTAL OF INFECTION.

He referred to the pharyngeal tonsil, the tubal tonsils, or cushion of the Eustachean tube, the faucial or true tonsil, and the glossic tonsil, after simply alluding to the anatomy. The physiology of this tissue was also alluded to, their importance as blood-making organs and the protection which they give the body in health by the filtration and attenuation of organisms.

The author then showed how these organs were constantly exposed to the pathogenic organisms found in liquids, air and food, and the nasal and buccal secretions. He referred to the works of Buschke, Schlenker, and others, to prove that pus-producing organisms may be absorbed from the tonsil. Referring to the toxic effects resulting from tonsillitis, he quoted many cases in the literature of sepsis and pyemia, in which the tonsil was proven the open gateway for the entrance of pyogenic organisms into the blood and tissue of the body. The relation of acute articular rheumatism and the allied diseases of endocarditis and chorea to tonsillitis, was clearly shown, as was the fact that diphtheria, scarlatina and other exanthemata may result from the absorption of microörganisms and their toxins from the diseased tonsil. The author also simply alluded to the fact that in some cases of typhoid fever in which no intestinal lesion had been found, the tonsil, by reason of its similarity in structure to Peyer's patches, may be the point of entry of the typhoid bacilli. The relation of scrofulosis and tuberculosis to diseased tonsillar tissue was explained.

The following conclusions were mentioned: (1) That the normal tonsil has a physiological function, probably protection to the organism; (2) that, being in itself often diseased, the physiological function is impaired and that, instead of being protective, it is the nidus for the growth and distribution of pathogenic organisms and their poisonous products into the system; (3) that many grave and fatal general infections have their origin in the tonsils; (4) that if the exanthemata, especially scarlatina, are of bacterial origin, the tonsil acts in part as a portal of entry; (5) that acute articular rheumatism, and the diseases often associated with it, endocarditis and chorea, in a great majority of cases are due to the action of attenuated bacteria, their toxins, or both, entering the general system through a diseased tonsil; (5) that in rare cases of typhoid fever in which no intestinal ulceration can be demonstrated, the similarity of the tonsillar tissue and Peyer's patches suggests the portal of entry to the typhoid bacillus in the tonsil; (7) that scrofulosis is often associated with diseased tonsillar tissue—mostly pharyngeal—and that the tubercle bacillus often enters the system via the tonsil; (8) that the tonsil is too little examined at necropsy and that much light might be shed on fevers of uncertain origin by the bacteriological and histological examinations.

DISCUSSION.

Dr. Allen A. Jones.—Dr. Ullman's valuable paper deals at length with, and calls our attention to, an important subject, that of infection by the tonsils. The study of the subject so far leads us to conclude that there are several diseases most commonly associated with initial tonsillar lesion, as, for instance, diphtheria, scarlet fever, and probably rheumatism. The association of follicular tonsillitis with typhoid fever, as seen in my case referred to by Dr. Ullman, I regard as a coincidence, although the throat affection showed not the slightest signs of yielding to treatment. Primary tuberculosis of the tonsil is a matter I would like to be further informed upon by some here present. Particular interest attaches to the question of the association of rheumatism and diseases of the tonsils. Personally, I have had my tonsils completely cut out in the hope that a subacute muscular rheumatism might thereby be prevented from recurring. I have, however, carefully studied several cases of rheumatism lately occurring in my practice, and in none of them was there any sore throat before, during, or after the rheumatic attack. The question is one that needs further careful study.

DeLancey Rochester.—The subject is very interesting. I rise to emphasise one point. Tonsils are not the portals of entry unless diseased. Diphtheria in many cases originates in the throat here more than the tonsil is involved and we have the systematic invasion from other regions than the tonsil; also a large number of cases originate in the nose and probably the disease occurs there more often than is suspected. After using antitoxin there often occurs a profuse discharge of membrane from the nose. We then know the disease existed there too. In the matter of rheumatism we are all interested but to discuss its nature is beyond the scope of the subject before us. I have one case of tuberculosis to report: a young man with cough; no evidence of diseased lung found upon examination of the chest; retained weight although not strong; hollow chest and stooping; both tonsils large; irritation in crypts probably increased by cough. I amputated both tonsils and cough subsided; within two weeks cough returned; within six months patient died of acute tuberculosis in both lungs. The tonsils probably were tuberculous. This makes me more careful about taking tonsils off with a knife. Probably other methods, as cautery and hot snare are better, leaving no surface for absorption.

Dr. E. A. Smith.—I want to ask if exposed to cold, a tonsil can take on a hyperemic condition and cause osteomyletis, just as arthritis will follow gonorrhea.

In closing the discussion, Dr. Ullman again emphasised the fact that the tonsil may be the seat of primary tuberculosis. He quoted

Schlesinger, who said that in general tuberculosis the tonsil is found tuberculous, and vice versa, that if the tonsil is tuberculous, there will often result tuberculosis elsewhere. He thoroughly agreed with Dr. Rochester that the tonsil, in order to be the portal of entry, must in itself be diseased and stated that this disease may only be the rare faction epithelium, so small as to be microscopical and that this may be the "locus minoris resistentiæ;" that there must however be a loss in the immunity of the individual, and that this accounts for the fact that many of these cases are ushered in by a cold, producing an active hyperemia of these susceptible parts. He agrees with Dr. Rochester that the nose is often affected in diphtheria and explained the susceptibility as due to the fact that there is no tonsillar tissue in the nose. Dr. Ullman answered the inquiry of Dr. Smith as to relations of osteomylitis and quoted Singer's paper on this subject, showing a relation between the two affections in the young. Manfredis's article was quoted, showing that tonsil like other lymphatic tissue had the power of attenuating bacteria.

---

*Section on Obstetrics and Gynecology, October 23, 1900.*

REPORTED BY WM. G. RING, M. D., Secretary.

The regular monthly meeting of this section was held at the Academy Rooms, Tuesday evening, October 23, 1900, at 8.30 p.m.

The meeting was called to order by the chairman, Dr. A. J. COLTON.

The minutes of the previous meeting were read and approved.

Dr. H. D. INGRAHAM read a paper on Personal experience with uterine fibroids.

Dr. C. E. CONGDON concurred with the essayist. The question whether to operate or not was debatable. When pregnancy is a present condition, and the tumor below the pelvic zone, operate; when above it, defer operation. The use of the curette is dangerous in fibroids, infection being apt to follow with disastrous results. I do not believe that there is any efficient medical treatment. Cannot say in the use of thyroids, what condition indicates their use. Perhaps Dr. Ingraham can indicate when and when not to use them.

Tait's operation is an ideal one in selected cases of fibroids, with most beautiful result, the tumors entirely disappearing, even when the size of an adult head.

Dr. Congdon then related the details of a case of supposed pregnancy of a period of two and a half months. The uterus was curetted, fetus removed. There still remained a nodule in the cavity of the uterus; this was removed with a placenta forceps and proved to be a small fibroma. He also had, ten days ago, what he supposed was a pedunculated fibroid; the fundus uteri felt depressed, cup-shaped; thought the uterus had become inverted. An abdominal section was made, a number of small fibroids of the fundus were found. A hysterectomy was done and recovery took place. Palliative treatment is not recommended except where operation is not feasible.

In closing the discussion Dr. Ingraham said about the use of thyroid, I would not give it to a woman who was weak or debilitated, or to one with a weak heart, otherwise I would give five grains a day, increasing the dose; it is not a reliable remedy.

Number present, 21. Meeting adjourned at 9.45.

---

### EARLY RECOGNITION OF CANCER OF THE UTERUS.

Humiston, (*Jour. Amer. Med. Assn.*, Vol 35, No. 13,) says it has been his experience that the most common form of cancer of the cervix, the adenocarcinoma, is by far the most to be dreaded:

1. Because of its natural growth in expanding laterally into the parametrium.

2. Because the lymphatic vessels and glands in the parametrium are so small that they may easily be overlooked, yet may be infected.

3. Because of the indirect connection to the chain of glands over the iliac vessels.

He argues for

1. Early differentiation of malignant growths of the cervix.

2. Careful consideration of the importance of the lymphatic vessels and glands in their capacity of drains of the different portions of the uterus.

3. That abdominal section with complete removal of these special groups of lymphatics alone offers in suitable cases the chance against a recurrence of carcinoma, particularly when the cervix is affected. —*Memphis Medical Monthly.*

